# The Apoptotic Effect of Ursolic Acid on SK-Hep-1 Cells is Regulated by the PI3K/Akt, p38 and JNK MAPK Signaling Pathways

**DOI:** 10.3390/molecules21040460

**Published:** 2016-04-20

**Authors:** Wan-Ling Chuang, Ping-Yi Lin, Hui-Chuan Lin, Yao-Li Chen

**Affiliations:** 1 Transplant Medicine & Surgery Research Centre, Changhua Christian Hospital, Changhua 50006, Taiwan; 159260@cch.org.tw (W.-L.C.); 69221@cch.org.tw (P.-Y.L.); 2 Department of Nursing, Hung Kuang University, Taichung 43302, Taiwan; 3 Department of Surgery, Changhua Christian Hospital, Changhua 50006, Taiwan; 4 School of Medicine, Kaohsiung Medical University, Kaohsiung 80708, Taiwan

**Keywords:** apoptosis, ursolic acid, SK-Hep-1

## Abstract

Ursolic acid (UA) is a pentacyclic triterpene acid that is present in a wide variety of medicinal herbs and edible plants. This study investigated the effect of UA on apoptosis and proliferation of hepatocellular carcinoma SK-Hep-1 cells. After treatment of SK-Hep-1 cells with different concentrations of UA, we observed that cell viability was reduced in a dose- and time-dependent manner. Furthermore, there was a dose-dependent increase in the percentage of cells in the sub-G1 and G2/M phases, with cells treated with 60 μM showing the highest percentages of cells in those phases. UA-induced chromatin condensation of nuclei was observed by using DAPI staining. The western blot results revealed that exposure to UA was associated with decreased expression of the anti-apoptotic proteins Mcl-1, Bcl-xL, Bcl-2, and TCTP and increased expression of apoptosis-related proteins TNF-α, Fas, FADD, Bax, cleaved caspase-3, caspase-8, caspase-9, and PARP. Immunocytochemistry staining showed that treatment with UA resulted in increased expression of caspase-3. Moreover, exposure to UA resulted in the inhibition of the PI3K/Akt and p38 MAPK signaling pathways. These findings suggest that UA inhibits the proliferation of SK-Hep-1 cells and induces apoptosis.

## 1. Introduction

**Figure 1:**
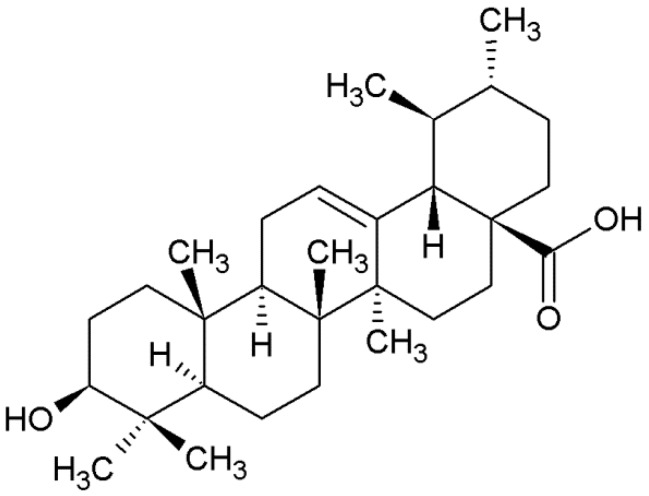
The structure of UA (3β-hydroxyurs-12-en-28-oic acid).

Ursolic acid (UA, 3β-hydroxyurs-12-en-28-oic acid, Figure 1) is a pentacyclic triterpenoid carboxylic acid found in abundance in a number of plants and medicinal herbs such as apples [1], basil, bilberries, cranberries, lavender, rosemary (*Rosmarinus officinalis* L.) [2], sage (*Slavia ofﬁcinalis* L.) [3], snake-needle grass (*Oldenlandia diffusa*) [4] and Jamun plum (*Eugenia jumbolana*) [5].

Studies have demonstrated that UA has a number of pharmacological effects including anti-inflammatory, anti-oxidative [6], antibacterial, antifungal [7], anti-mutagenic, antiangiogenic [8], anti-viral, anticarcinogenic [9], hepatoprotective [10], anti-atherosclerotic anti-tumor [11] and anti-hyperlipidemic activity. UA has also been shown to induce apoptosis in a number of human cancer cell lines [5,12,13,14,15,16,17,18,19,20]. 

Hepatocellular carcinoma (HCC) is one of the most common malignant tumors and is the second leading cause of cancer death worldwide [21]. According to BCLC staging system, the treatment methods for HCC were different depending on staging [22]. Although numerous treatment methods have been suggested for HCC management, such as surgical resection and liver transplantation, nevertheless, the 5-year survival rate is lower than 70% in patients with early HCC who have received surgical resection [23]. Thus, it is imperative to find some novel drugs to treat HCC. 

Apoptosis, or programmed cell death, plays an important role in many physiological processes, such as tissue development and maintenance of homeostasis [24]. In mammals, the initiation of apoptosis is governed by two major pathways: an intrinsic pathway and an extrinsic pathway. The intrinsic (mitochondria-associated) apoptotic pathway is regulated by the Bcl-2 family of proteins. The extrinsic pathway (also known as death receptor pathway) is another apoptotic pathway and mediated via death receptors including tumor necrosis factor (TNF) receptor-1, CD95/Fas receptors, TRAIL receptor-1, and TRAIL receptor-2. Death receptor-mediated extrinsic pathway by activing downstream effectors involves caspases 3, 8 and 10 in inducing apoptosis [25]. Therefore, finding novel drugs target for inducing apoptosis in cancer cells could contribute to the development of new anti-cancer agents [26].

In this study, we examined the effect of UA on apoptosis and cell proliferation in the human hepatocellular carcinoma cell line SK-Hep-1. We found that treatment with UA inhibits the proliferation of SK-Hep-1 cells and induces apoptosis by inhibition of the PI3K/Akt and p38 MAPK signaling pathway. 

## 2. Results

### 2.1. Cytotoxic Effect of UA on SK-Hep-1 Cells

**Figure 2:**
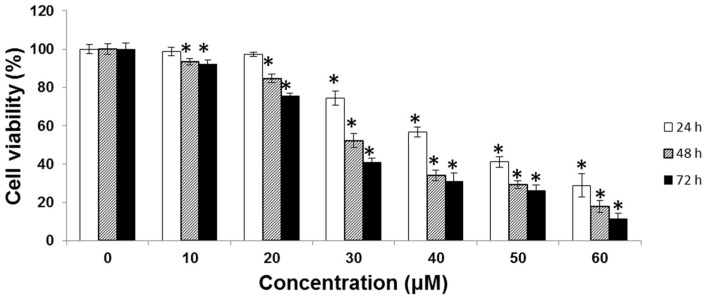
SK-Hep-1 cells (2 × 10^4^ cells/well) were treated with various concentrations of UA (0, 10, 20, 30, 40, 50, and 60 μM) for 24, 48, or 72 h. Cell viability was measured using the MTT assay. The cytotoxicity of UA in SK-Hep-1 cells was dose-dependent. Each point is the mean ± SD of three experiments. * indicates significant difference from control, *p* < 0.001.

SK-Hep-1 cells were treated with various concentrations (0, 10, 20, 30, 40, 50, and 60 μM) of UA for 24, 48, and 72 h, and cell viability was measured using the MTT assay. The half-maximal inhibitory concentration (IC_50_) was 52.8 μM at 24 h, 40.7 μM at 48 h and 36.9 μM at 72 h (Figure 2). 

**Figure 3:**
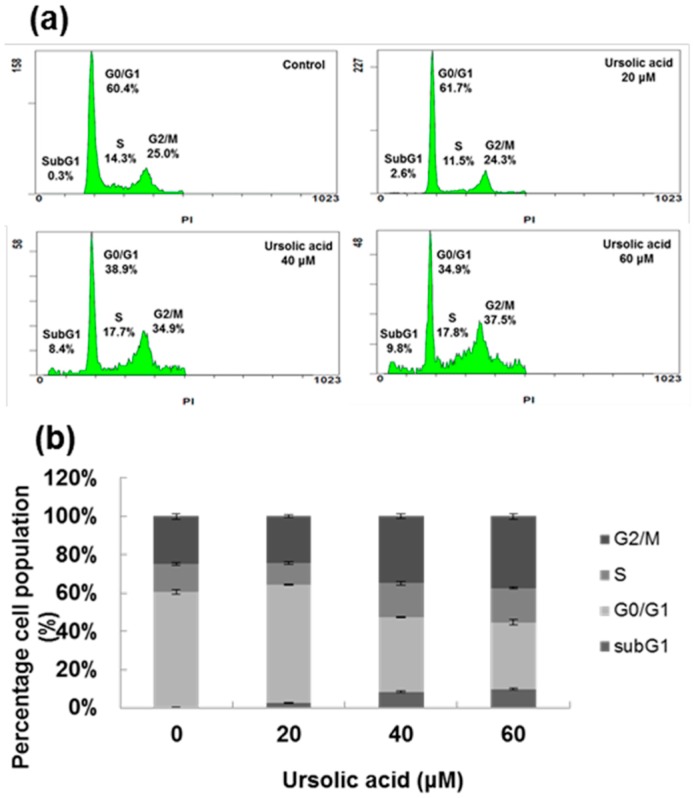
Effect of UA on the cell cycle of SK-Hep1 cells. (**a**) SK-Hep1 cells were treated with 0, 20, 40, and 60 μM UA for 24 h, after which the cells were stained with PI and analyzed by FACS; (**b**) Quantitative results of cell cycle percentages. FACS, fluorescence-activated cell sorting.

**Figure 4:**
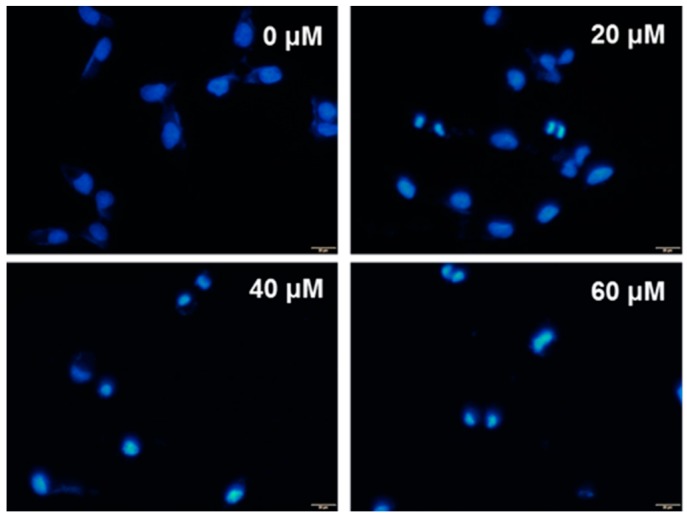
DAPI staining showing the effect of UA on chromatin condensation in SK-Hep-1 cells after treatment with 0, 20, 40, and 60 μM UA for 24 h. Images were obtained with an Olympus IX81 microscope.

The cell cycles were analyzed by fluorescence activated cell sorting (FACS). As seen in Figure 3, there was a dose-dependent increase in percentage of cells in the sub-G1 and G2/M phases, with cells treated with 60 μM showing the highest percentages of cells in those phases. The FACS results demonstrate that treatment with UA induces apoptosis. In addition, UA induced chromatin condensation of nuclei was observed by using DAPI staining (Figure 4).

### 2.2. Immunocytochemical Analysis 

**Figure 5:**
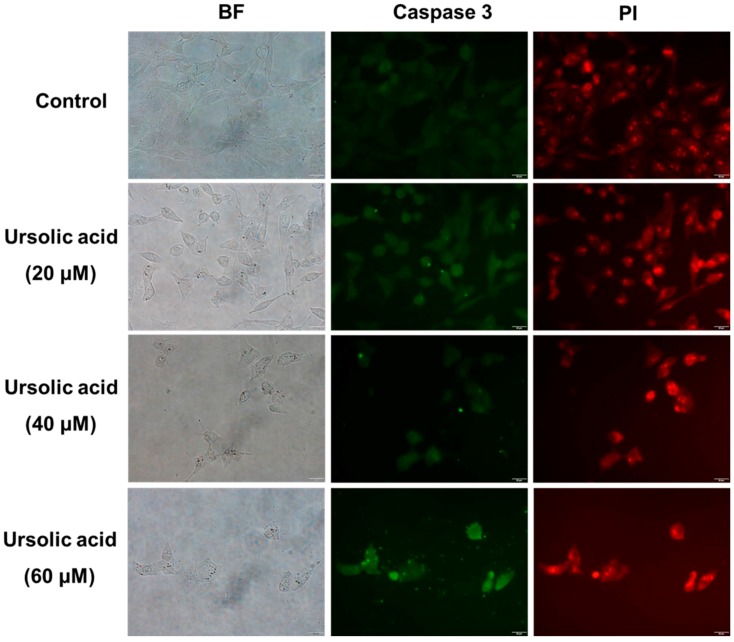
Immunocytochemical analysis. SK-Hep-1 cells were treated with 0, 20, 40, and 60 μM UA for 24 h and then fixed with 4% paraformaldehyde to allow for detection by caspase 3 antibody. The results revealed that treatment with UA for 24 h resulted in increased expression of caspase-3.

SK-Hep-1 cells were treated with various concentrations (0, 20, 40, and 60 μM) of UA for 24 h and then fixed with 4% paraformaldehyde to allow for the detection of caspase-3 by staining with antibodies. SK-Hep-1 cells treated with UA had increased expression of caspase-3 at 24 h (Figure 5), suggesting that UA induces apoptosis, at least in part, by promoting the expression of caspase-3 in SK-Hep-1 cells in a dose-dependent manner. 

### 2.3. UA Induces Expression of TNF-α, Fas, FADD, Bax, Bcl-xL and Inhibits Expression of Mcl-1, TCTP, and Bcl-2 Proteins in SK-Hep-1 Cells

SK-Hep-1 cells were treated with various concentrations (0, 20, 40, and 60 μM) of UA for 24 h and the relative expression of apoptosis-related proteins was evaluated by western blotting. 

**Figure 6:**
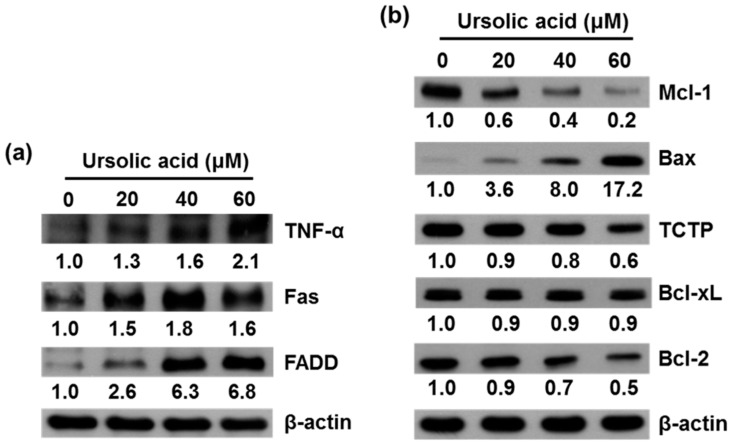
The effects of UA on protein expression of TNF-α, Fas, FADD, Mcl-1, Bax, TCTP, Bcl-2, and PARP in SK-Hep-1 cells. SK-Hep-1 cells were treated with UA (0, 20, 40, and 60 μM) for 24 h and protein expression was evaluated by western blotting. The results showed that UA resulted in increased protein expression of (**a**) TNF-α, Fas and FADD; (**b**) decreased expression of Mcl-1, TCTP, and Bcl-2, and increased protein expression of Bax and Bcl-xL. β-Actin served as a loading control.

The results demonstrated significantly higher levels of TNF-α, Fas, FADD (Figure 6a), and Bax (Figure 6b) protein expression and significantly lower levels of Mcl-1, TCTP and Bcl-2 protein expression in cells treated with UA than in untreated controls. There were no significant differences in level of Bcl-xL protein expression between treated and untreated cells (Figure 6b).

### 2.4. Effect of UA on Activation of Caspases and PARP

SK-Hep-1 cells were treated with various concentrations (0, 20, 40, and 60 μM) of UA for 24 h and the relative expression of apoptosis-related proteins was evaluated by western blotting. 

**Figure 7:**
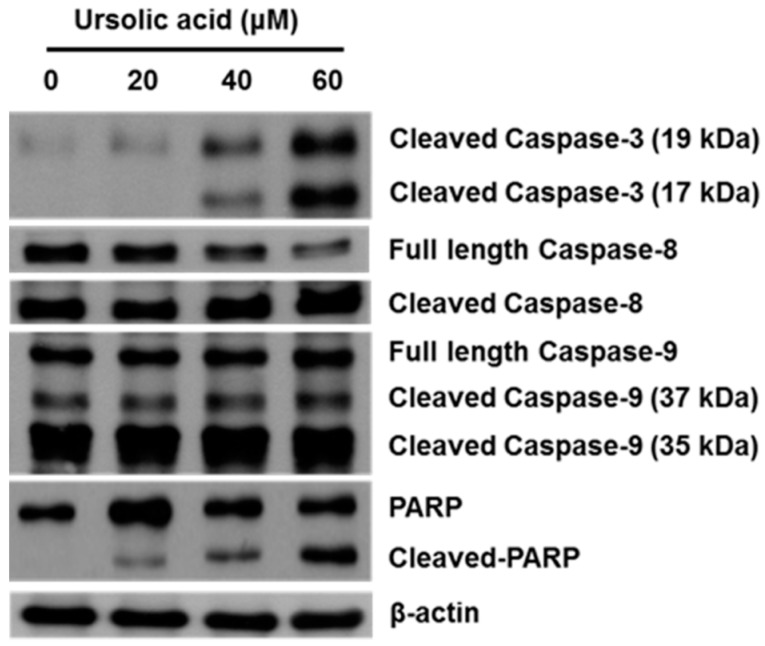
Activation of caspase-3, caspase-8, caspase-9, and PARP protein expression in SK-Hep-1. SK-Hep-1 cells were treated with UA (0, 20, 40, and 60 μM) for 24 h and the protein expression was evaluated by western blotting. The results showed that UA treatment resulted in increased protein expression of cleaved caspase-3, cleaved caspase-8, cleaved caspase-9, and cleaved PARP. β-Actin served as a loading control.

We found that the levels of expression of cleaved caspase-3, caspase-8, caspase-9, and PARP were significantly higher in cells treated for 24 h with UA than in untreated controls (Figure 7). 

### 2.5. UA Induces Apoptosis in SK-Hep-1 Cells by Upregulating the PI3K/Akt, ERK1/2, JNK1/2 and p38 MAPK Pathways

**Figure 8:**
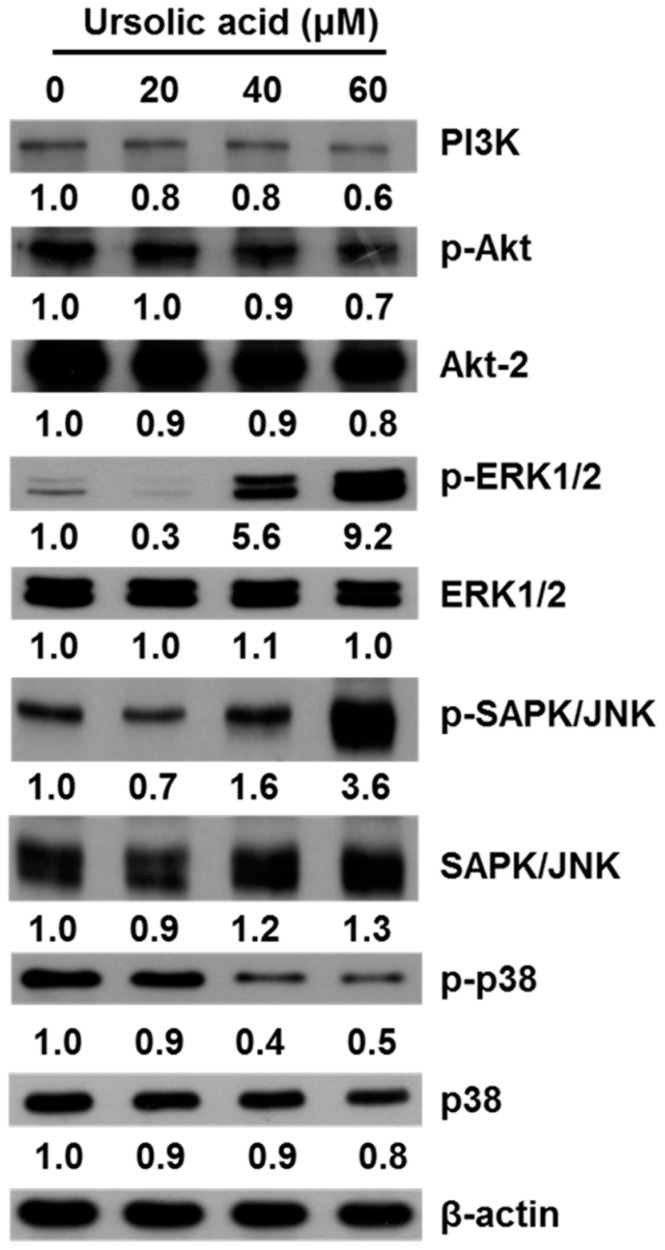
Effects of UA on the MAPK pathway and the PI3K/Akt pathway in SK-Hep-1 cells. The cells were treated with various concentrations of UA (0, 20, 40, and 60 μM) for 24 h. The levels of phosphorylation of PI3K, Akt, ERK1/2, JNK1/2, and p38 were detected by western blot analysis. β-Actin served as a loading control.

The mitogen-activated protein kinase (MAPK) pathway, which includes the signaling molecules ERK, JNK and p38, plays a critical role in cell proliferation, growth, differentiation, migration, and apoptosis [27]. To examine the molecular mechanisms of UA on apoptosis in SK-Hep-1 cells, we used western blot to detect the expression of PI3K, p-Akt, Akt-2, p-ERK1/2, ERK1/2, p-JNK, JNK, p-p38, and p38. We found that cells treated with various concentrations of UA (20, 40, and 60 μM) for 24 h had significantly higher levels of p-ERK1/2 and p-JNK protein expression than untreated controls and decreased the level of PI3K, p-Akt and p-p38 (Figure 8).

## 3. Discussion

UA is a pentacyclic triterpene acid that is present in a wide variety of medicinal herbs and edible plants. Several studies have shown that UA induces apoptosis in various human cancer cell lines, such as liver cancer cell lines (HepG2, Hep3B, Huh7 and HA22T) [5], gastric cancer cell lines (SNU-484) [13], gallbladder carcinoma cell lines (GBC-SD and SGC-996) [12], colon cancer cells (HT-29) [15], melanoma cells (B16F-10) [16], the human cervical carcinoma cancer cell line HeLa [17], the lung cancer cell line A549 [14], the human prostate cancer cell line PC3 [18], the human endometrial cancer cells (SNG-II and HEC108) [19] and pancreatic cancer clles (MIA PaCa-2 and PANC-1) [20]. UA also can inhibited cell growth and proliferation of Jurkat leukemic T-cells [28]. These studies show that UA is anti-proliferative and cytotoxic to different tumor cells. Apart from its anti-proliferative and cytotoxic effects on various tumor cells, the beneficial effects of UA on hepatic cells also have been reported. A previous study has suggested that UA could inhibit the development of non-alcoholic fatty liver disease by reducing endoplasmic reticulum stress [29]. Another study showed that UA inhibited the proliferation in hydrogen peroxide-induced malignant transformation rat hepatic oval cell line WB-F344, but had no inhibitory effect on the growth of rat hepatocyte cell line (BRL) was observed [30].

Our results show that UA inhibited the proliferation of SK-Hep-1 cells in a dose-dependent manner (Figure 2), with IC_50_ values of 52.8 μM at 24 h, 40.7 μM at 48 h and 36.9 μM at 72 h. It has been reported that treatment with osthole could induce G2/M arrest and apoptosis in lung cancer A549 cells [31,32]. We found that in the SK-Hep-1 cells treated with UA, there was a dose-dependent increase in sub-G1 and G2/M phases (Figure 3). In addition, UA-induced chromatin condensation of nuclei was observed by using DAPI staining (Figure 4). Our data indicate that UA inhibits the proliferation of SK-Hep-1 cells.

Apoptosis is a programmed cell death, and studies have shown that induction of apoptosis may be a potential treatment option for malignant diseases [25,33]. There are two main apoptosis signaling pathways. In the extrinsic pathway, initiated by death receptor (Fas receptor (CD95) and TNF receptor-1), activation of the downstream cell signaling transduction leads to induced cell apoptosis. For example, UA induces apoptosis via down-regulation of XIAP and mitochondrial-dependent pathway in human liver cancer HepG2, Hep3B, Huh7 and HA22T cell lines [5]. Our data show that treatment with UA was associated with increased protein expression of TNF-α, Fas, and FADD (Figure 6a) in SK-Hep-1 cells. 

The Bcl-2 family plays an important role in the control of apoptosis in many cell types [34]. The Bcl-2 family can be divided into two classes, including antiapoptotic proteins (Bcl-2, Bcl-xL, Bcl-w and Mcl-1) and proapoptotic proteins (Bax, Bad, Bak, Bik, and Bid) [24]. We found that UA treatment resulted in increased expression of Bax protein and decreased expression of Mcl-1, TCTP and Bcl-2 (Figure 6b). UA induced apoptosis via activation of caspases in human liver cancer HepG2, Hep3B, Huh7 and HA22T cell lines [5]. We observed the activation of both caspases-3 and -8 that indicated the caspases’ function in the extrinsic induced apoptosis. There are a number of signaling pathways that can induce apoptosis, including the phosphatidylinositol 3-kinase (PI3K)/Akt pathway [35] and the mitogen-activated protein kinase (MAPK) pathway. The MAPK family, including extra-cellular signal-regulated kinase (ERK), Jun kinase (JNK/SAPK), and p38 MAPK are known to play important roles in cell viability and apoptosis [36]. The growth of cells is regulated through the PI3K/Akt pathways [37]. It has been reported that inhibition of the PI3K/Akt pathway could induced cell death [38]. Down-regulation of survival and activation of caspase-3 through the PI3K/Akt pathway by UA induced HepG2 cell apoptosis [5]. Our results thus indicate that UA induces cell death by inhibiting the activation of the PI3K/Akt pathway in SK-Hep-1 cells (Figure 8). We also found that ERK and JNK activation participate in the regulation of apoptosis. The present study demonstrates that UA induces apoptosis in SK-Hep-1 cells by activating the JNK1/2 pathway (Figure 8).

## 4. Materials and Methods

### 4.1. Chemical Reagents 

MTT [3-(4,5-dimethylthiazol-2-y1)-2,5-diphenyltetrazolium bromide] and dimethyl sulfoxide (DMSO) were obtained from Merck (Darmstadt, Germany). Paraformaldehyde, Triton X-100, β-actin and propidium iodide (PI) were obtained from Sigma Chemical Co. (St. Louis, MO, USA). Dulbecco’s Modified Eagle Medium (DMEM), fetal bovine serum (FBS), 10× phosphate-buffered saline (PBS), and penicillin-streptomycin were obtained from Gibco BRL (Grand Island, NY, USA) and 4′6-diamidino-2-phenylindole (DAPI) was obtained from Invitrogen (Carlsbad, CA, USA). 10× RIPA Lysis Buffer was obtained from Millipore (Billerica, MA, USA). Tween 20 was obtained from Amresco (St. Louis, MO, USA) and WesternBright Quantum ECL HRP substrate was obtained from Advansta (Menlo Park, CA, USA). The Bax, Bcl-xL, Fas, FADD, Mcl-1, TCTP, caspase-3, caspase-9, PARP, PI3K, phospho-Akt, Akt, phospho-ERK1/2, ERK1/2, phospho-SAPK/JNK, SAPK/JNK, phospho-p38, and p38 antibodies were all obtained from Cell Signaling Technology Inc. (Beverly, MA, USA), and TNF-α, Bcl-2, caspase-8 antibodies were obtained from Novus Biologicals (Littleton, CO, USA). 

### 4.2. Cell Culture

The human liver carcinoma cell line SK-Hep-1 was obtained from the Bioresource Collection and Research Center (Hsinchu, Taiwan). The cells were maintained in DMEM medium with 10% FBS, 100 units/mL penicillin, and 0.1 mg/mL streptomycin at 37 °C in a humidified atmosphere of 95% air and 5% CO_2_.

### 4.3. Preparation of UA 

UA of over 98% purity was purchased from Santa Cruz Biotechnology (Santa Cruz, CA, USA). Empirical formula: C_30_H_48_O_3_; molecular weight: 456.70. Melting point 285 °C. 100 mM stock solution of UA was prepared and stored at −20 °C until required. 

### 4.4. Cell Viability Assay

Cell viability was assessed using the MTT assay. SK-Hep-1 cells were plated at a density of 2 × 10^4^ cells/well in 96-well plates and incubated overnight. After removing the MEM-α medium, the cells were treated with various concentrations (10, 20, 30, 40, 50, and 60 μM) of UA for 24, 48, and 72 h. After treatment, cells were treated with MTT (1 mg/mL) and incubated for 2 h at 37 °C. The medium was removed and the purple-blue MTT formazan precipitate was dissolved in 100 μL of DMSO. Absorbance was measured at 590 nm in a microplate reader (Thermo Multiskan SPECTRUM, Thermo Fisher Scientific, Waltham, MA, USA). Results are expressed as a percentage of the untreated controls. The rate of proliferation was calculated with the following formula:

Proliferation (%) = (OD_test_ − OD_blank_) × 100, (1)

where OD_test_ and OD_blank_ are the optical density of the test substances and the blank controls, respectively.

### 4.5. Cell Cycle Analysis

SK-Hep-1 cells were treated with various concentrations (0, 20, 40, and 60 μM) of UA for 24 h, and then collected, centrifuged and fixed with ice-cold ethanol (70%) overnight at −20 °C. The cell pellets were then treated with propidium iodide (PI) solution (containing 100 μg/mL RNase) for 30 min at 37 °C. Subsequently, the samples were analyzed by a Cytomics™ FC500 flow cytometer (Beckman Coulter, Miami, FL, USA). A minimum of 10,000 cells were analyzed to determine DNA content, and the percentage of cells in each cell cycle phase was quantified.

### 4.6. Immunocytochemical Staining 

SK-Hep-1 cells were treated with various concentrations (0, 20, 40, and 60 μM) of UA for 24 h, after which they were washed with ice-cold PBS and then fixed with 4% paraformaldehyde in PBS for 30 min at 37 °C. Cells were then washed twice with ice-cold PBS and then incubated in 0.25% Triton X-100 in 0.1% BSA for 5 min at 4 °C. Cells were then washed twice with ice-cold PBS and then incubated with PBS containing 0.1% BSA for 1 h at room temperature to block non-specific binding. Thereafter, the cells were separately incubated with rabbit anti-caspase 3 (1:200) antibody in PBS containing 0.1% BSA overnight at 4 °C. After washing three times with PBS the cells were incubated with anti-rabbit FITC (1:500) in PBS containing 0.1% BSA for 1 h at room temperature. After washing three times with PBS, the nuclei were stained with 5 μg/mL PI. After staining, the samples were immediately examined under an Olympus IX81 microscope (Olympus, Tokyo, Japan).

### 4.7. DAPI Staining Analysis

SK-Hep-1 Cells (2 × 10^5^) were plated onto 6-well plates and treated with various concentrations of UA (0, 20, 40, and 60 μM) for 24 h. The cells were then fixed with 4% paraformaldehyde for 30 min at room temperature and then washed twice with PBS. Cells were then permeabilized in 0.25% Triton-X 100 for 5 min at 4 °C and then washed three times in PBS. The nuclei were stained with 1 μg/mL DAPI for 10 min in the dark and the cells were imaged with a fluorescence microscope (Olympus IX81).

### 4.8. Cell Lysis and Western Blot Analysis

After UA treatment, SK-Hep-1 cells were washed with ice-cold PBS and then lysed in ice-cold whole cell extract buffer containing protease inhibitors. Cells were scraped and collected into eppendorf tubes, which were then vibrated for 30 min at 4 °C, followed by centrifugation at 13,000× *g* for 10 min at 4 °C. Protein concentrations were determined using a BCA protein assay kit (Thermo, Rockford, IL, USA). Equal amounts (10 μg/lane) of sample were loaded into wells comprising 6%–10% polyacrylamide gel and then separated using SDS-PAGE. Separated proteins were then electrophoretically transferred to polyvinylidene difluoride membranes (PVDF, Millipore) at 400 mA for 2 h. Membranes were then incubated in blocking buffer (PBS with 0.05% tween 20 and 0.1% BSA) for 1 h at room temperature and then incubated with primary antibodies overnight. Membranes were then incubated with secondary antibodies conjugated with horseradish peroxidase. Blots were washed three times in 1× PBS-Tween solution and incubated for 1 min with ECL reagents. The results were visualized by exposing blots to super RX-N film (Fujifilm Corporation, Tokyo, Japan). The antibodies used in this study included TNF-α, Fas, FADD, Mcl-1, TCTP, Bcl-2, caspase-3, caspase-8, caspase-9, PARP phospho-ERK1/2, ERK1/2, phospho-SAPK/JNK, SAPK/JNK, phospho-p38 and p38.

### 4.9. Statistical Analyses

Data are expressed as the mean ± standard deviation and were compared using the Student’s *t*-test. A *p* value of <0.05 was considered to indicate statistical significance. All statistical analyses were performed using GraphPad Prism software, version 4.0 (GraphPad Software, Inc., La Jolla, CA, USA).

## 5. Conclusions 

Taken together, our study demonstrated that UA induces apoptosis by regulating the PI3K/Akt and p38/JNK MAPK signaling pathways and activation of the extrinsic and intrinsic apoptosis signaling pathways in human SK-Hep-1 cells. Therefore, UA may be a potentially useful chemotherapeutic agent for HCC.

## Abbreviations

PARP: poly (ADP-ribose) polymerase
MAPK: mitogen-activated protein kinase
PI3K: phosphoinositide 3-kinase
ERK: extracellular signal-regulated kinase
JNK: c-Jun N-terminal kinase
p38: p38 MAP kinase
